# Comparative analysis of biofilm detection methods and antibiotic resistance in catheter-associated uropathogens: a cross-sectional study from Syria

**DOI:** 10.1186/s12866-025-04506-2

**Published:** 2025-11-17

**Authors:** Walid Khaddam, Bushra Durgham

**Affiliations:** Department of Biochemistry and Microbiology, Faculty of Pharmacy, Homs University, Homs‎, Syria

**Keywords:** Biofilm, Catheter-Associated urinary tract infection (CAUTI), Microplate assay, Antibiotic resistance, Uropathogens

## Abstract

Catheter-associated urinary tract infections (CAUTIs) are a major healthcare challenge due to bacterial biofilm formation, which protects pathogens from antibiotics and host immune responses. Three phenotypic biofilm detection methods—Microplate assay, Tube Method, and Modified Congo Red Agar (MCRA)— were compared using bacterial isolates from catheter tips and urine samples. The Microplate assay, considered the reference standard, detected biofilm in 88.6% of catheter isolates and 78.6% of urine isolates. Notably, 44% of urine samples showed no microbial growth, likely due to prior antibiotic use. In catheter-derived samples, CRA showed higher sensitivity (81.8%) and specificity (61.5%) than the Tube method (72.7% and 46.2%, respectively). PPV and NPV were 87.0% and 46.2% for CRA, and 82.2% and 22.7% for Tube. Both methods performed less reliably in urine isolates. Strong biofilm formation was more prevalent in catheter isolates (62.5%) than in urine isolates (44.6%) and was associated with higher antimicrobial resistance. Gentamicin was most effective against urine isolates (85.7%), whereas Imipenem showed highest efficacy in catheter isolates (47.7%). These findings provide practical guidance for microbiology laboratories, especially in low-resource settings, by identifying reliable phenotypic methods for biofilm screening. Overall, sensitive biofilm detection combined with targeted antibiotic susceptibility testing is crucial for effective CAUTI management and antimicrobial stewardship.

## Introduction

Biofilms are structured communities of microorganisms embedded within a self-produced extracellular polymeric substance (EPS), known as the biofilm matrix, which enables microbial cells to adhere to each other as well as to biotic and abiotic surfaces [1, 2]. Biofilm formation is a dynamic, multi-stage process, generally progressing through initial reversible adhesion, irreversible adhesion, microcolony formation, maturation, and eventual dispersion [3, 4].

Within these biofilms, microbial cells exhibit physiological and metabolic traits distinct from their planktonic counterparts. The biofilm matrix—composed of exopolysaccharides, proteins, lipids, extracellular DNA, and water—constitutes the majority of the biofilm volume, providing protection against antibiotics, host immune responses, and environmental stress, while facilitating quorum-sensing communication [2, 3, 5]. Gram-negative bacteria typically use acyl-homoserine lactones, whereas Gram-positive bacteria rely on oligopeptides to coordinate essential biofilm functions, including nutrient acquisition, maturation, virulence factor expression, and horizontal gene transfer of antibiotic resistance genes [5, 6]. Enzymes such as dispersin B and nucleases mediate matrix degradation, enabling biofilm dispersal and colonization of new sites, which can lead to persistent and recurrent infections [3, 7].

Clinically, biofilms contribute to a broad spectrum of infections, ranging from dental plaque and gingivitis to prosthetic device-related infections and chronic lung infections in cystic fibrosis patients [4, 8]. Indwelling medical devices (IMDs), including urinary catheters and central venous lines, are particularly prone to biofilm colonization, accounting for 60–70% of device-associated nosocomial infections [9, 10]. Catheter-associated urinary tract infections (CAUTIs) are among the most frequent healthcare-associated infections, with biofilms developing on urinary catheters within 3–4 days and contributing to infection in up to 25% of catheterized patients [2, 11]. These multispecies biofilms display strong resistance to antibiotics, complicating treatment and promoting the emergence of multidrug-resistant organisms [12, 13]. Given the clinical relevance of biofilms in CAUTIs and their role in antimicrobial resistance, early and accurate detection of biofilm-producing bacteria is essential. Therefore, The aim of this study was to identify biofilm-forming bacteria from patients with CAUTIs and evaluate their antibiotic susceptibility profiles, providing insights for effective infection management.

## Materials and methods

### Sample collection

A total of 100 hospitalized patients who had undergone urinary catheterization for at least two days were enrolled, as prolonged catheterization is a recognized risk factor for catheter-associated urinary tract infections (CAUTIs) [14, 15]. Two types of specimens were collected from each patient under aseptic conditions: Midstream urine samples were aspirated directly from the catheter tubing using a sterile syringe. Samples were immediately transported to the microbiology laboratory and processed within two hours to minimize bacterial overgrowth or loss of viability. Catheter tip segments (2–3 cm in length) were collected from the distal end of the catheter. The presence of biofilm deposits was assessed macroscopically by trained personnel, and only segments with visible biofilm were included in biofilm-specific analyses. Catheter segments were handled under sterile conditions to prevent contamination. Samples without visible biofilm were excluded from biofilm detection analyses but were included in microbial distribution analyses. All samples were transported in sterile containers and processed promptly to ensure accuracy and minimize contamination [15].

### Demographic and clinical data

Demographic and clinical information was recorded for all participants. The study population included 54 males (54%) and 46 females (46%). Regarding age distribution, 70% of patients (*n* = 70) were older than 65 years, 20% (*n* = 20) were between 50 and 65 years, and 10% (*n* = 10) were younger than 50 years. All patients were hospitalized in the same clinical setting, providing a uniform source population. This demographic profile reflects the predominance of elderly patients among CAUTI cases, which is relevant for interpreting microbiological and clinical findings.

### Inclusion and exclusion criteria

Inclusion criteria: Adult patients (≥ 18 years) hospitalized and catheterized at Al-Zahraa and Al-Ahli Hospitals in Homs, Syria, who provided urine or catheter samples for routine culture during the study period. Only bacterial isolates were included for biofilm formation assessment and antibiotic susceptibility testing.

Exclusion criteria: Samples showing no bacterial growth or containing non-bacterial organisms (e.g., Candida spp., other fungi) were excluded. Samples with incomplete clinical data or those improperly collected or handled were also excluded from the analysis.

### Ethical considerations

The research was conducted in accordance with institutional and national ethical guidelines and the principles of the Declaration of Helsinki. Ethical approval was obtained from the local ethics committees of Al-Zahraa and Al-Ahli Hospitals, Homs, Syria. The ethics committee waived the requirement for written consent due to the observational nature of the study, and verbal informed consent was obtained from all patients or their legal guardians prior to sample collection. Patient confidentiality and anonymity were strictly maintained.

### Microbiological processing

Urine samples were cultured on cysteine lactose electrolyte-deficient (CLED) agar, MacConkey agar, and blood agar plates, followed by incubation at 37 °C for 24 h under aerobic conditions [16]. The catheter segments were first rinsed with sterile distilled water to remove loosely attached cells. The outer surfaces were then disinfected with 70% ethanol to eliminate planktonic organisms. Using sterile surgical scissors, the catheter tips were cut into 4–5 fragments and incubated in nutrient broth for 24 h at 37 °C to enrich bacterial growth [15]. After incubation, the broth cultures were streaked using a calibrated loop onto CLED, MacConkey, and blood agar plates, which were then incubated at 37 °C for another 24 h. Bacterial isolates were identified based on colony morphology, Gram staining characteristics, and a series of standard biochemical tests including catalase, oxidase, and triple sugar iron (TSI) test, among others as appropriate [16].

### Microbiological processing

Urine samples were cultured on cysteine lactose electrolyte-deficient (CLED) agar, MacConkey agar, and blood agar plates, followed by incubation at 37 °C for 24 h under aerobic conditions [16].

Catheter segments were first rinsed with sterile distilled water to remove loosely attached cells. The outer surfaces were then disinfected with 70% ethanol to eliminate planktonic organisms. Using sterile surgical scissors, catheter tips were cut into 4–5 fragments and incubated in nutrient broth for 24 h at 37 °C to enrich bacterial growth [15]. After incubation, the enriched broth cultures were streaked onto CLED, MacConkey, and blood agar plates using a calibrated loop, and incubated again at 37 °C for 24 h.

Bacterial isolates were identified based on colony morphology, Gram staining, and standard biochemical tests, including catalase, oxidase, and triple sugar iron (TSI) tests, among others, as appropriate [16].

### Antibiotic susceptibility testing

All bacterial isolates were tested for antibiotic susceptibility using the Kirby-Bauer disk diffusion method on Mueller-Hinton agar plates, following the Clinical and Laboratory Standards Institute (CLSI) guidelines [17]. Briefly, bacterial suspensions were adjusted to the 0.5 McFarland standard, swabbed uniformly onto Mueller-Hinton agar plates, and antibiotic discs were applied to the surface. Plates were incubated at 37 °C for 18–24 h, after which the inhibition zones were measured. Results were interpreted according to CLSI criteria. The antibiotics tested included: Ceftazidime (30 µg), Amoxicillin-Clavulanate (Augmentin, 30 µg), Cefotaxime (30 µg), Cefepime (30 µg), Levofloxacin (15 µg), Piperacillin-Tazobactam (85 µg), Sulfamethoxazole-Trimethoprim (25 µg), Cefuroxime (30 µg), Gentamicin (10 µg), Amikacin (30 µg), Imipenem (10 µg), and Nitrofurantoin (300 µg) [18].

### Detection of biofilm production

Biofilm production by isolated uropathogens was assessed using three phenotypic methods: the Tube method, Congo Red Agar method, and Tissue Culture Plate method [19]. These methods are widely used for qualitative and quantitative evaluation of biofilm formation in clinical microbiology.

### Tissue culture plate method (TCPM)

The Tissue Culture Plate Method (TCPM) was used as the gold standard for detecting biofilm formation. A loopful of freshly cultured bacterial isolates was inoculated into 10 mL of trypticase soy broth supplemented with 1% glucose. After incubation, 180 µL of sterile trypticase soy broth was dispensed into separate wells of a sterile 96-well flat-bottom polystyrene tissue culture plate, and 20 µL of the bacterial suspension was added, resulting in a 1:10 dilution. The plate was covered with Parafilm and incubated at 37 °C for 24 h [20]. After incubation, the plate was gently shaken, and the contents were discarded to remove planktonic cells. Each well was then washed three to four times with sterile distilled water and inverted to dry. Biofilms were fixed by adding 200 µL of 2% sodium acetate for 30 min. The wells were washed again three to four times with sterile distilled water, followed by staining with 200 µL of 0.1% crystal violet for 15 min. The plate was washed similarly and left to dry inverted [21]. After drying, the optical densities (OD) of the stained biofilms were measured at 570 nm using a micro-ELISA reader. Each test was performed in triplicate, and the average OD was calculated. The degree of biofilm formation was classified based on OD values relative to the control (Odc) as follows (Table 1).

**Table 1:** Grading of biofilm formation by uropathogenic isolates using microplate (Tissue culture Plate) method

Classification	Criteria/OD range
Non-biofilm producer	OD ≤ Odc
	(OD ≤ 0.37)
Weak biofilm producer	Odc < OD ≤ 2 Odc
	(0.38 < OD ≤ 0.74)
Moderate biofilm producer	2 Odc < OD ≤ 4 Odc
	(0.74 < OD ≤ 1.48)
Strong biofilm producer	OD > 4 Odc
	(OD > 1.48)

*OD* Optical density measured at 570 nm, *Odc* Optical density of negative control (sterile broth)

### Replicates and variability

Each assay was performed in triplicate for each isolate. The mean OD of the three replicates was calculated for classification. Variability across replicates was assessed by calculating the standard deviation (SD). Isolates with high variability (> 15% of the mean OD) were retested to confirm the consistency and reliability of the results.

### Tube method

A loopful of bacterial isolates from overnight cultures was inoculated into glass tubes containing 10 mL of trypticase soy broth supplemented with 1% glucose. The inoculated tubes were incubated at 37 °C for 24 h. After incubation, the broth was discarded, and the tubes were gently washed with sterile distilled water and allowed to dry. The dried tubes were stained with crystal violet for 15 min, followed by rinsing with sterile distilled water to remove excess stain. The tubes were then inverted and examined for biofilm formation. A visible film lining the bottom and walls of the tube indicated positive biofilm production, whereas the formation of a ring of stain at the air-liquid interface was considered a negative result [22].

### Modified congo red agar method (MCRA)

The conventional Congo Red Agar (CRA) method utilizes a standard agar medium (brain–heart infusion) supplemented with 0.8 g/L Congo red and 5–10 g/L sucrose to detect biofilm formation, where black, dry, crystalline colonies indicate biofilm production, while red or pink colonies indicate non-biofilm producers. However, CRA performance can vary across bacterial species, particularly Gram-positive isolates, and may yield false-negative results.

 In this study, we employed a Modified CRA (MCRA) protocol to enhance sensitivity and reproducibility. The blood agar base was supplemented with 0.4 g/L Congo red and 10 g/L glucose, and then sterilized by autoclaving at 121 °C for 15 min. Isolated uropathogens were inoculated onto MCRA plates and incubated aerobically at 37 °C for 24 h. Black, dry, crystalline colonies were interpreted as biofilm producers, whereas pink or red colonies were considered non-producers [23]. To minimize interpretation bias, all plates were read independently by two trained microbiologists, and any discrepancies were resolved by a third observer. This approach accounted for inter-species differences in colony morphology and reduced false-negative results, particularly for Gram-positive bacteria.

### Statistical analysis

Data were analyzed using SPSS software, version 25.0 (IBM Corp., Armonk, NY, USA). Categorical variables were presented as frequencies and percentages, and comparisons were performed using the chi-square test or Fisher’s exact test, as appropriate. Continuous variables were expressed as mean ± standard deviation (SD) and compared using the Student’s t-test or one-way ANOVA, as applicable. A p-value of < 0.05 was considered statistically significant.

## Results

### Distribution of bacterial isolates in urine and catheter samples

**Fig. 1:**
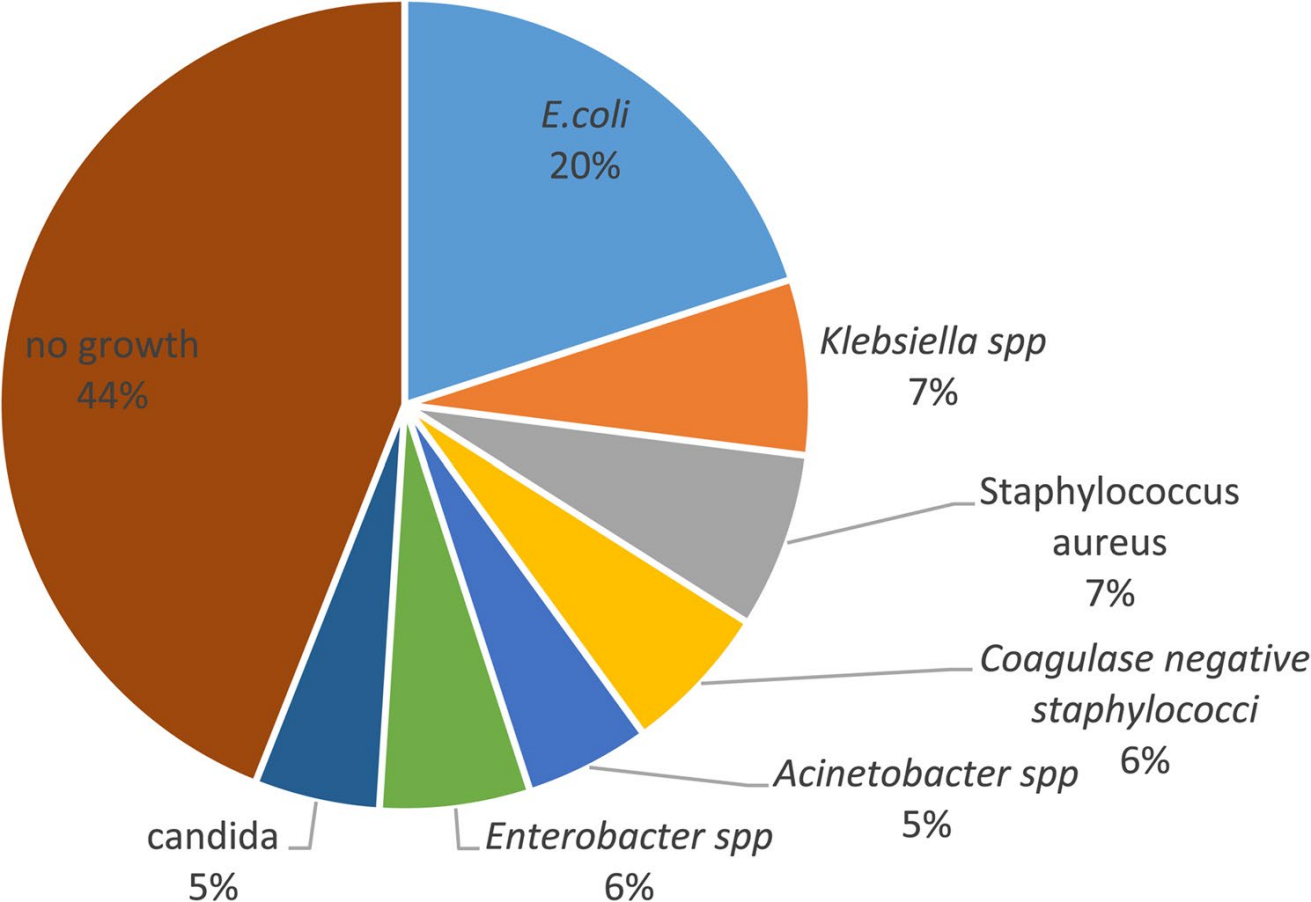
Distribution of bacterial isolates from urine samples (*n* = 100) collected from catheterized patients. Isolates were identified using standard culture and biochemical methods (CLED, MacConkey, and blood agar), following CLSI guidelines. Percentages indicate the proportion of each species among all urine samples, including culture-negative cases

A total of 100 urine samples and 100 catheter samples were analyzed. In urine samples, *E. coli* was the most frequently isolated organism (20/100, 20%), followed by *Klebsiella spp.* (7/100, 7%) and *coagulase-negative Staphylococci* (7/100, 7%). *Coagulase-positive Staphylococci* and *Enterobacter spp.* were each detected in 6/100 (6%) samples, while *Acinetobacter spp.* and *Candida spp.* were isolated in 5/100 (5%) of cases. No microbial growth was observed in 44/100 (44%) of urine samples, likely due to prior antibiotic therapy or low bacterial load **(**Fig. 1**).**

**Fig. 2:**
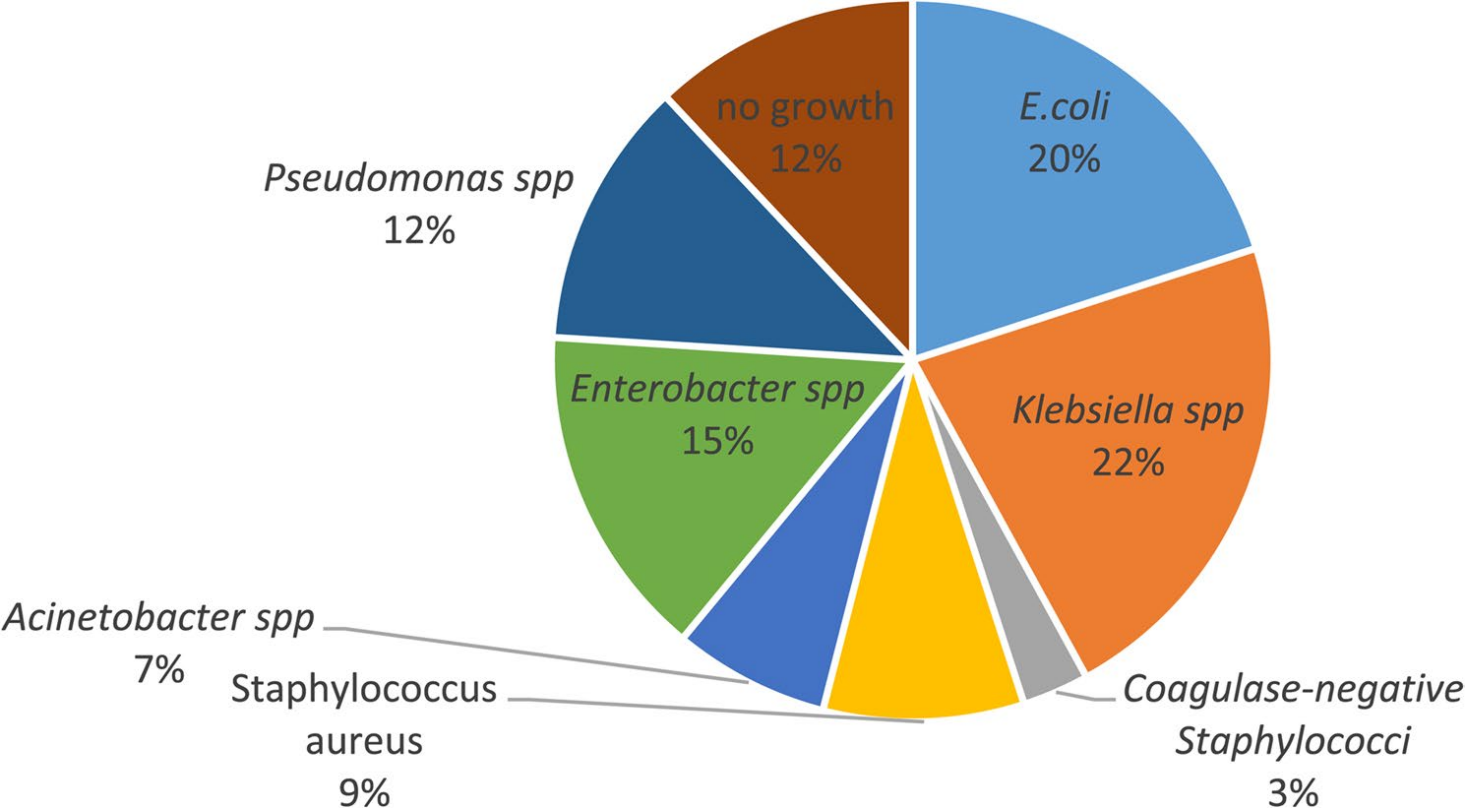
Distribution of bacterial isolates from catheter tip segments (*n* = 100) obtained from the same patient cohort. Identification was performed by culture on CLED, MacConkey, and blood agar followed by biochemical tests. Percentages represent species distribution among all catheter samples

In catheter samples, *Klebsiella spp.* was the predominant isolate (22/100, 22%), followed by *E*. *coli* (20/100, 20%) and *Enterobacter spp.* (15/100, 15%). *Pseudomonas spp.* accounted for 12/100 (12%) of isolates and was exclusively detected in catheter samples, likely reflecting its enhanced adhesion to catheter surfaces. *Coagulase*-*positive Staphylococci* and *Acinetobacter spp.* were found in 9/100 (9%) and 7/100 (7%) of catheter samples, respectively, while *coagulase-negative Staphylococci* were less frequent (3/100, 3%). Only 12/100 (12%) of catheter samples showed no microbial growth **(**Fig. 2**)**. Indicating that catheter tip cultures may be more sensitive in detecting colonization compared to urine samples.This variation in microbial profiles between the two groups highlights the dynamic nature of pathogen distribution in catheter-associated infections and may be influenced by differences in patient characteristics, catheterization duration, or sample type.

### Comparison of biofilm formation detection methods in urinary and catheter isolates

The diagnostic performance of the Congo Red Agar (CRA) and Tube method for detecting biofilm formation in catheter and urine isolates was evaluted, using the Microplate assay as the reference standard.

In catheter-derived samples, the CRA method demonstrated superior diagnostic performance, with a sensitivity of 81.8%, specificity of 61.5%, and overall accuracy of 75.6%. In contrast, the Tube method showed lower sensitivity (72.7%), specificity (46.2%), and accuracy (68.9%). Chi-square analysis revealed a statistically significant correlation between both CRA and Tube methods and the Microplate assay (*p* < 0.05), with CRA exhibiting stronger agreement. Furthermore, CRA demonstrated a high positive predictive value (PPV) of 87.0%, indicating reliable identification of true biofilm-positive cases, whereas its negative predictive value (NPV) was relatively low (46.2%), suggesting limited reliability in excluding biofilm formation. The Tube method had a slightly lower PPV (82.2%) and much lower NPV (22.7%), indicating a higher likelihood of false negatives.

In urine samples, both methods showed reduced diagnostic performance. CRA had a sensitivity of 78.4% and specificity of 43.8%, with an overall accuracy of 70.2%. Its PPV decreased to 67.8%, and NPV was 38.9%. The Tube method showed the weakest performance, with sensitivity and specificity of 64.7% and 25%, respectively, accuracy of 55.2%, PPV of 60.0%, and a very low NPV of 18.2%. Chi-square analysis did not reveal a statistically significant correlation between either method and the Microplate assay in urine samples (*p* ≈ 0.08 for CRA, *p* > 0.4 for Tube).The lower predictive performance in urine isolates may be attributed to reduced bacterial load, prior antibiotic exposure, or less dense biofilm formation compared to catheter surfaces **(**Table 2**).**

**Table 2:** Shows the sensitivity, specificity, accuracy, and p-value values ​​for catheter and urine samples

Sample Type	Method	Sensitivity (%)	Specificity (%)	Accuracy	PPV (%)	NPV (%)	*P*-Value
Catheter	mCRA	81.8	61.5	75.6	87.0	46.2	< 0.05
	TM	72.7	46.2	68.9	82.2	22.7	< 0.05
Urine	mCRA	78.4	43.8	70.2	67.8	38.9	> 0.05
	TM	64.7	25.0	55.2	60.0	18.2	> 0.05

### Classification of uropathogenic isolates according to biofilm formation

The biofilm-forming capacity of bacterial isolates was evaluated using three distinct methods—Microplate (optical density measurement), Tube method, and Congo red agar (CRA)—for catheter-derived (*n* = 88) and urine-derived (*n* = 56) isolates. Each isolate was classified into four categories based on biofilm intensity: strong, moderate, weak, and negative.

**Fig. 3:**
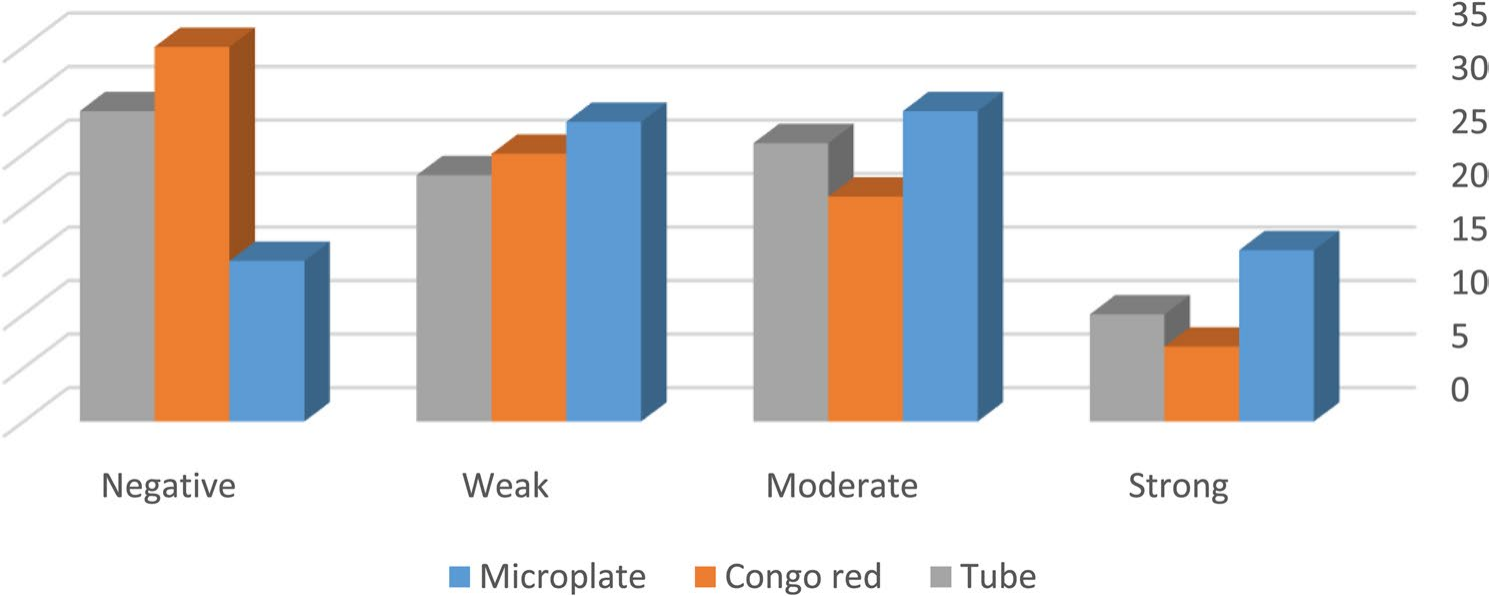
Distribution of catheter-derived isolates (*n* = 88) by biofilm formation strength, classified using Microplate Assay optical density (OD) as the reference method, and comparative results from Tube Method ™ and Modified Congo Red Agar (mCRA). Biofilm classification: Strong = positive by all three methods; Moderate = positive by two methods; Weak = positive by one method; Negative = no positivity by Microplate assay. OD thresholds for Microplate assay: ≤0.37 = non-biofilm, 0.38–0.74 = weak, 0.75–1.48 = moderate, > 1.48 = strong. Values shown as percentages of total catheter isolates

For catheter isolates, the Microplate assay identified 16 isolates (18.2%) as strong biofilm producers, 29 isolates (33.0%) as moderate, 28 isolates (31.8%) as weak, and 15 isolates (17.0%) as negative. Using CRA, 7 isolates (8.0%) were strong, 21 (23.9%) moderate, 25 (28.4%) weak, and 35 (39.8%) negative. The Tube method classified 10 isolates (11.4%) as strong, 26 (29.5%) as moderate, 23 (26.1%) as weak, and 29 (33.0%) as negative. These results demonstrate the Microplate method’s superior sensitivity in detecting even weak biofilm formation on catheter surface. (Fig. 3)

**Fig. 4:**
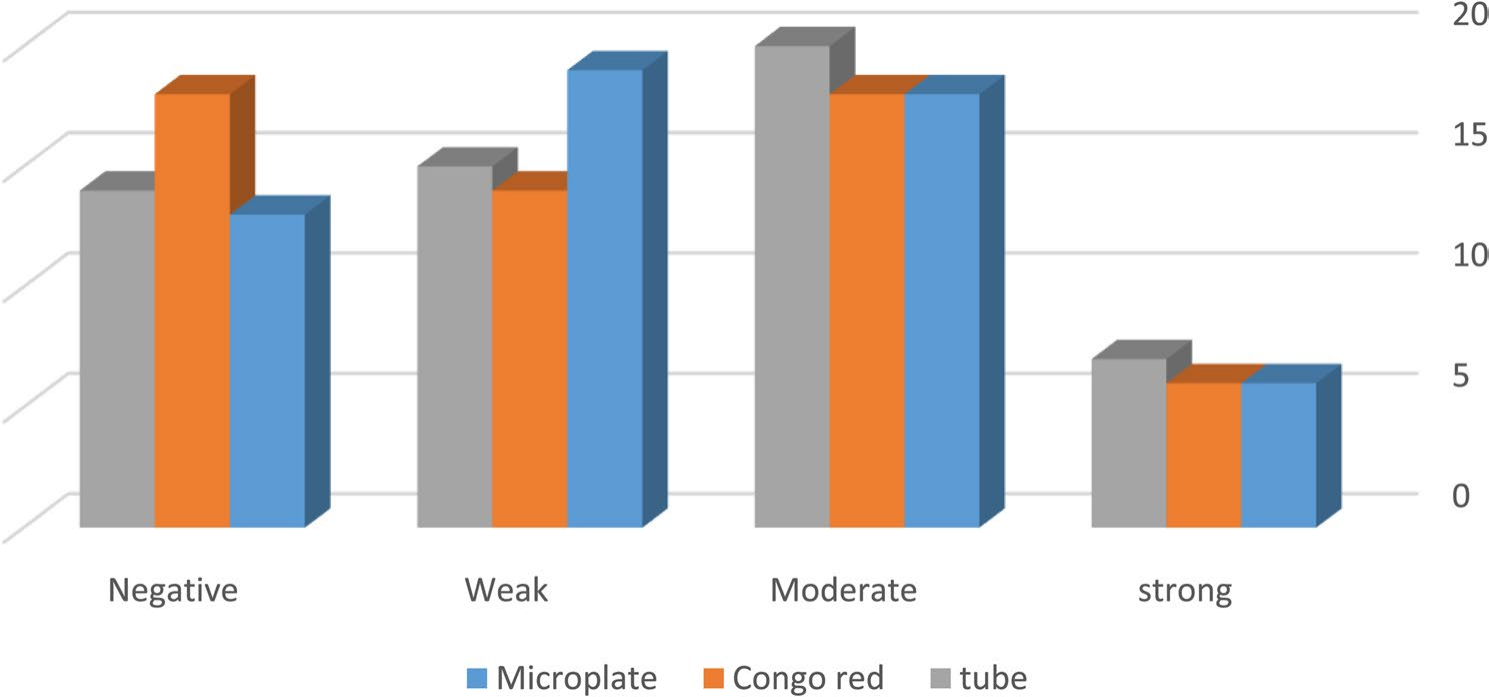
Distribution of urine-derived isolates (*n* = 56) by biofilm formation strength, classified as described in Fig. 3.OD-based Microplate assay served as the reference standard, and results were compared with Tube Method and Modified Congo Red Agar (mCRA). Values shown as percentages of total urine isolates

For urine isolates, the Microplate method detected 11% of isolates as strong, 32% as moderate, 33.8% as weak, and 23.2% as negative. CRA results were slightly more conservative, with 11% strong, 32% moderate, 25% weak, and 32% negative. The Tube method identified 13% strong, 36% moderate, 27% weak, and 25% negative isolates. Overall, these findings indicate variability in detection performance across the three methods, with the Microplate assay showing higher sensitivity for weak and moderate biofilm producers, while the Tube and CRA methods tended to classify fewer isolates as strong. **(**Fig. 4**)**

The lower detection rates in urine isolates may reflect reduced bacterial load, prior antibiotic exposure, or less dense biofilm formation compared to catheter surfaces. These results highlight the importance of using multiple complementary methods to accurately assess biofilm formation, particularly in clinical settings with low bacterial densities, and provide practical guidance for laboratories, including those in low-resource settings.

### Antimicrobial resistance patterns of urinary and catheter isolates

A marked disparity was observed in antimicrobial resistance between isolates from urine samples and those from indwelling urinary catheters. Resistance rates were significantly higher among catheter-associated isolates for nearly all tested antibiotics.

For urine isolates (*n* = 56), resistance rates were as follows: Ceftazidime (CAZ) 20/56 (35.7%), Amoxicillin-Clavulanate (AUG) 18/56 (32.1%), Levofloxacin (LEV) 25/56 (44.6%), Trimethoprim-Sulfamethoxazole (SXT) 16/56 (28.6%), Gentamicin (CN) 8/56 (14.3%), and Imipenem (IMP) 8/56 (14.3%).

For catheter isolates (*n* = 88), resistance rates were substantially higher: Ceftazidime 80/88 (90.9%), Amoxicillin-Clavulanate 85/88 (96.6%), Levofloxacin 84/88 (95.5%), Trimethoprim-Sulfamethoxazole 84/88 (95.5%), Gentamicin 40/88 (45.5%), and Imipenem 46/88 (52.3%).

### Species-wise resistance profiles

Urine isolates: *E. coli* (*n* = 20) and *Klebsiella spp*. (*n* = 14) displayed the highest resistance, ranging from 20% to 60%. *Enterobacter spp.* (*n* = 6) and *Acinetobacter spp.* (*n* = 5) showed intermediate resistance (30–50%), while *Staphylococcus spp*. (*n* = 13) exhibited the lowest rates (10–30%).

**Fig. 5:**
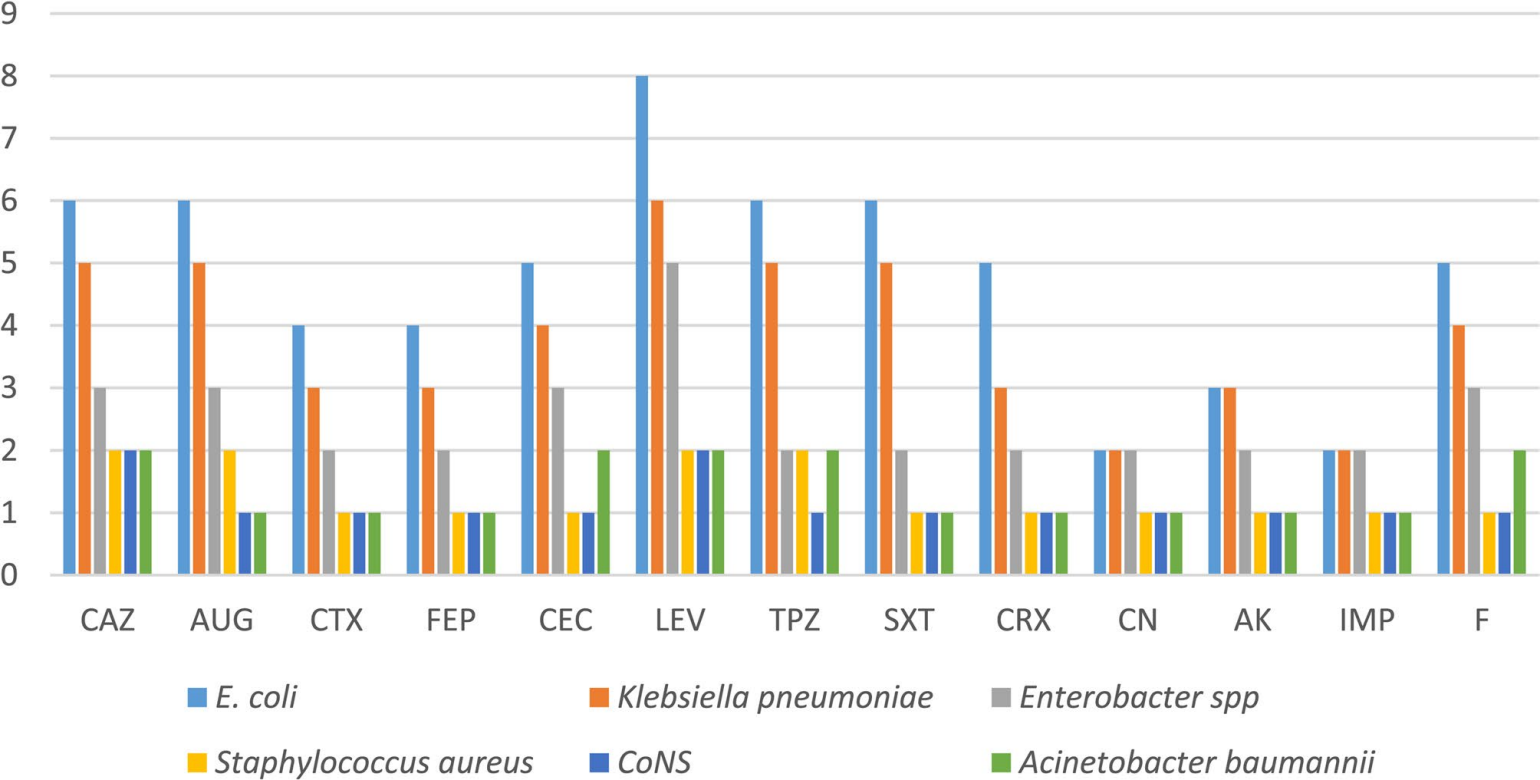
Antimicrobial resistance profiles of urine isolates (*n* = 56) against tested antibiotics, determined using the Kirby-Bauer disk diffusion method on Mueller-Hinton agar, following CLSI 2022 guidelines. Bars represent the percentage of isolates resistant to each antibiotic

Catheter isolates: *Klebsiella spp*. (*n* = 22) exhibited resistance > 80%, followed by *E*. *coli* (*n* = 20), *Enterobacter spp*. (*n* = 15), and *Acinetobacter spp*. (*n* = 7) with rates of 75–90%. *Pseudomonas spp*. (*n* = 12), exclusively isolated from catheters, showed resistance between 70 and 90%. *Staphylococcus spp*. in catheter samples (*n* = 12) demonstrated moderate resistance (30–50%), lower than most Gram-negative organisms (Fig. 5).

The observed higher resistance in catheter isolates correlates with the greater prevalence of strong biofilm formation in these samples (62.5% vs. 44.6% in urine isolates). Biofilms protect bacterial cells from antibiotics and host defenses, contributing to chronic infection and treatment failure. Notably, although Imipenem showed the lowest resistance among catheter isolates (52.3%), the rate remains clinically significant, emphasizing the need for culture-guided therapy rather than empirical use of broad-spectrum agents. Gentamicin retained good activity against urine isolates, making it a valuable option for uncomplicated UTIs, whereas Imipenem may be reserved for confirmed multidrug-resistant CAUTI cases.

Statistical analysis using Chi-square or Fisher’s exact test confirmed that resistance rates were significantly higher in catheter isolates compared to urine isolates for most antibiotics (*p* < 0.05).

**Fig. 6:**
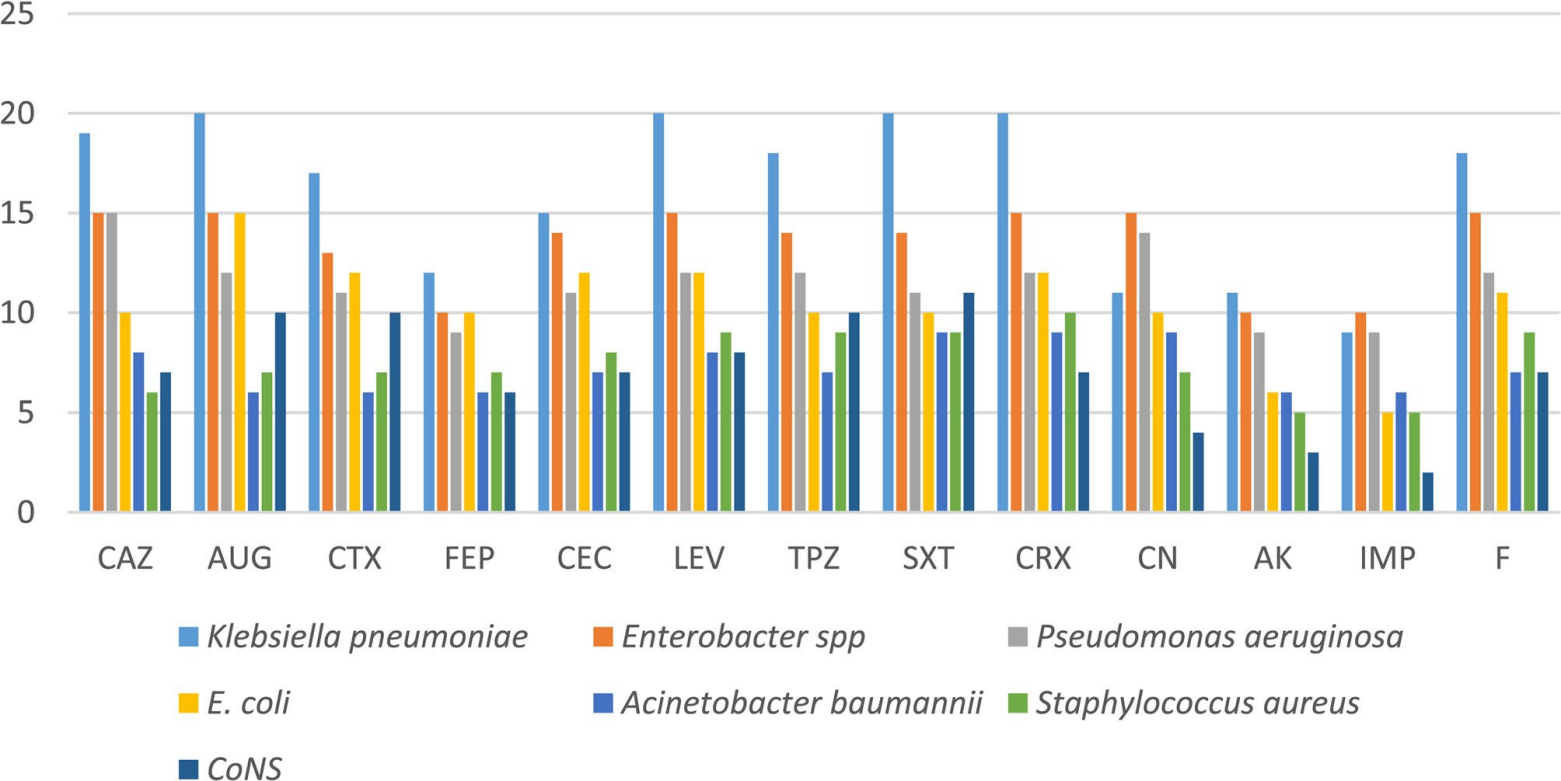
Antimicrobial resistance profiles of catheter isolates (*n* = 88) against tested antibiotics, determined using the Kirby-Bauer disk diffusion method on Mueller-Hinton agar, following CLSI 2022 guidelines. Bars represent the percentage of isolates resistant to each antibiotic

Gentamicin was significantly more effective in urine isolates than Levofloxacin (*p* = 0.0009), Ceftazidime (*p* = 0.0164), and Amoxicillin-Clavulanate (*p* = 0.0440). No significant difference was observed between Gentamicin and broad-spectrum agents like Imipenem or Amikacin. In catheter isolates, Imipenem showed significantly lower resistance compared to Cefotaxime, Ceftazidime, Levofloxacin, AUG, and SXT (*p* < 0.001). No significant differences were observed between Imipenem and Amikacin or Cefepime (Fig. 6).

## Discussion

The comparative analysis of biofilm detection methods revealed that the Microplate assay remains the most accurate and reliable standard for identifying biofilm-forming bacterial isolates in both catheter and urine samples. Among the alternative techniques, the Congo Red Agar (CRA) method consistently outperformed the traditional Tube method in terms of sensitivity, accuracy, and positive predictive value, particularly in catheter samples [24, 25]. In catheter isolates, the CRA method showed significantly higher diagnostic performance, with a sensitivity of 81.8% and accuracy of 75.6%. This finding suggests a strong potential for CRA in reliably identifying biofilm-producing organisms in catheter-associated infections, where biofilm formation plays a central role [26]. By comparison, the Tube method demonstrated lower sensitivity (72.7%) and accuracy (68.9%), and a notably lower negative predictive value (22.7%), limiting its clinical utility in ruling out biofilm presence [27].

The enhanced performance of the CRA method can be attributed to the use of a modified CRA protocol, which improves contrast and stability of the medium, facilitating better differentiation between biofilm-positive and negative isolates [28]. These modifications are particularly beneficial in detecting moderate biofilm producers, which are often overlooked by the Tube method due to its reliance on subjective visual scoring and semi-quantitative nature [29]. However, the diagnostic performance of both CRA and Tube methods declined in urine samples. Neither method demonstrated statistically significant agreement with the Microplate reference, nor both showed low negative predictive values, suggesting a high rate of false negatives. This reduced performance may result from differences in microbial density, presence of inhibitors in urine, or reduced biofilm biomass in non-catheterized environments [30, 31].

Importantly, while CRA demonstrated higher PPV and sensitivity, the relatively low NPV values across both sample types highlight a key limitation: a negative result by CRA or Tube does not reliably exclude biofilm formation. Therefore, reliance solely on these simpler methods may result in underdiagnosis, particularly in clinically suspected cases where accurate detection is critical for guiding therapy [32]. In summary, the CRA method is a more effective alternative to the Tube method, especially when used with a modified protocol. However, due to their limited ability to exclude biofilm-negative cases, confirmatory testing using the Microplate method is recommended whenever possible, particularly in catheter-associated infections. This approach ensures greater diagnostic accuracy and informs appropriate therapeutic strategies, ultimately improving clinical outcomes [33, 34].

Comparative evaluation of biofilm detection methods demonstrated that the Microplate assay provides the highest sensitivity and reliability for detecting biofilm-forming bacteria in both catheter and urine samples. Its superior performance, evidenced by higher detection rates (88.6% in catheter samples and 78.6% in urine samples), reaffirms its status as the gold standard for biofilm assessment [35, 36]. In contrast, both the Tube and CRA methods exhibited lower sensitivity, particularly in urine samples, highlighting their limitations when used as standalone diagnostic tools [37].

Stratification of isolates according to biofilm production strength provided further insights into the pathogenic potential of catheter-associated versus urine-derived organisms. A significantly higher proportion of catheter isolates exhibited strong biofilm formation (62.5%) compared to urine isolates (44.6%) [38, 39]. This observation aligns with the well-established role of urinary catheters as surfaces that facilitate microbial adhesion, colonization, and the establishment of mature biofilms. Persistent exposure of catheters to urine flow, host proteins, and immune components may enhance microbial adaptation and promote more robust biofilm development [40]. Conversely, the weaker biofilm-forming profile observed in urine isolates suggests a less conducive environment for stable biofilm formation, likely due to transient bacterial presence and the lack of a solid substrate for long-term colonization [41]. Nonetheless, the presence of weak or moderate biofilm producers in urine samples still underscores the potential clinical relevance of these organisms, especially in recurrent or persistent urinary tract infections (UTIs) [42].

A system for grading biofilm formation intensity was applied based on concordance among the three diagnostic methods (Microplate, CRA, and Tube). Samples were categorized as strong (positive by all three methods), moderate (positive by two methods), weak (positive by one method), and negative (negative by the Microplate reference method) (Table 3). This classification provides a more nuanced assessment than the conventional binary approach, reflecting the consistency of biofilm detection across different methodologies. Although not commonly reported in the literature, it offers valuable insights into the robustness of biofilm production and may help differentiate isolates with stable, strong biofilm-forming ability from those with less consistent or weak formation. Such stratification can enhance diagnostic reliability and has potential clinical implications, as stronger biofilm formation is often associated with increased antimicrobial resistance and infection severity. Further studies are warranted to validate the clinical relevance and predictive value of this classification system, potentially establishing it as a useful tool in both research and clinical diagnostics.

**Table 3:** Shows the classification of urine and catheter isolates according to the strength of biofilm formation

Sample Type	Biofilm Formation Strenght	Number of Sampels	Proportion (%)
Urine (56)	Strong (Positive in all 3 methods)	25	44
	Moderate (Positive in 2 methods)	15	26.8
	Weak (Positive in 1 method)	11	19.6
	Negative (No positivity by Microplate)	5	8.9
Catheter (88)	Strong (Positive in all 3 methods)	55	62.5
	Moderate (Positive in 2 methods)	16	18.2
	Weak (Positive in 1 method)	12	13.6
	Negative (No positivity by Microplate)	5	5.7

The observed variation in bacterial distribution between urine and catheter samples may be explained by several clinical and methodological factors. The higher rate of *Klebsiella spp*. and *Pseudomonas spp*. in catheter isolates is consistent with their well-known ability to adhere to catheter surfaces and form biofilms, which enhances persistence and colonization compared to urine samples. In contrast, the predominance of *E. coli* in urine specimens aligns with its role as the most frequent causative agent of community- and hospital-acquired urinary tract infections. The absence of microbial growth in a substantial proportion of urine samples (44%) could be attributed to prior administration of antibiotics before sampling, which likely reduced bacterial recovery. Additionally, the lower percentage of negative cultures in catheter samples (12%) suggests that catheter tips may provide a more sensitive medium for detecting colonization, even in cases where urine cultures fail. These findings highlight the importance of analyzing both urine and catheter specimens to obtain a more accurate picture of the microbial spectrum in catheter-associated urinary tract infections (CAUTIs).

The results also highlight the considerable difference in antimicrobial resistance between urine and catheter isolates. Catheter-associated isolates consistently demonstrated higher resistance, a trend likely linked to the formation of bacterial biofilms within the catheter environment. Biofilms are known to protect bacteria from host defenses and antibiotic action, leading to chronic infection and treatment failure [43, 44]. The high resistance levels observed in key pathogens such as Klebsiella spp., E. coli, and Enterobacter spp. In catheter samples suggest that biofilm formation plays a critical role in resistance development [45, 46]. The detection of *Pseudomonas spp*. exclusively in catheter samples further supports the hypothesis that the catheter environment supports colonization by more resistant and opportunistic organisms [47].

Despite the generally high resistance rates, Gentamicin retained significant activity against urine isolates, making it a valuable first-line agent for uncomplicated UTIs—especially considering its cost-effectiveness and narrower spectrum, which helps reduce selective pressure on broader agents [48]. Imipenem, on the other hand, maintained comparatively lower resistance rates in catheter isolates, positioning it as a key option for treating complicated infections like CAUTIs [49]. However, overreliance on broad-spectrum antibiotics like Imipenem poses a risk for accelerating resistance. Therefore, antibiotic selection should be guided by culture results and local resistance profiles. Whenever possible, narrower-spectrum yet effective agents like Gentamicin should be prioritized for uncomplicated cases, while broad-spectrum agents should be reserved for confirmed multidrug-resistant infections [50].

These findings underscore the importance of incorporating routine susceptibility testing and biofilm assessment in clinical diagnostics to optimize antimicrobial therapy and improve patient outcomes—particularly in settings with high catheter usage [51, 52].

## Conclusion

The finding demonstrates that biofilm formation plays a key role in antimicrobial resistance among catheter-associated urinary tract infections (CAUTIs). The Microplate assay was the most sensitive and reliable method for detecting biofilm-producing bacteria, outperforming both the Tube and Congo Red Agar methods. Catheter isolates exhibited a higher prevalence of strong biofilm producers and greater antibiotic resistance compared to urine isolates, highlighting the pathogenic potential of indwelling devices. These findings underscore the importance of routine biofilm detection and targeted antimicrobial susceptibility testing to guide effective treatment, improve patient outcomes, and support evidence-based infection control policies. Additionally, the study provides valuable local data on uropathogens, informing region-specific strategies. Future research should focus on developing rapid, cost-effective biofilm detection methods and innovative strategies to disrupt biofilms, thereby enhancing the management and control of CAUTIs. [53–58]

## Data Availability

The data that support the findings of this study are available from the corresponding author upon reasonable request. Due to patient confidentiality, data are not publicly available.

## Declarations

### Limitations

This study has several limitations, including its single-center design and relatively small sample size, which may limit generalizability. Biofilm detection was based on phenotypic methods that are inherently subjective and may vary between observers. Molecular confirmation of isolates was not performed, which might underestimate biofilm producers. Clinical outcomes (e.g., infection duration and treatment response) were not assessed, limiting correlations between biofilm formation, antimicrobial resistance, and patient outcomes. Future multicenter studies incorporating molecular and quantitative techniques are recommended.
